# Assessing recovery-related behaviors, emotions, and cognitions among members of alcoholics anonymous over 7 days: Quantitative daily diary data

**DOI:** 10.1016/j.dib.2020.105983

**Published:** 2020-07-03

**Authors:** Onawa LaBelle, Maurissa Hastings

**Affiliations:** University of Windsor, Windsor N8S1A1, ON, Canada

**Keywords:** Alcoholics anonymous, Helping behavior, Gratitude, Self-esteem, Affect, Recovery capital, Narcissism, Daily diary methodology, Time-based sampling

## Abstract

This was a quantitative daily dairy study that consisted of an initial baseline assessment followed by 7 nightly reports collected each evening. Participants were members of Alcoholics Anonymous (*N* = 113) and were recruited through social media networks (e.g., large recovery-related Facebook groups, twitter, Instagram, etc.), an email list from people in recovery who had previously participated in research, and through the use of snowball sampling. The analyses used in the resultant article was multilevel modeling with daily reports nested within individuals (LaBelle, 2020). These data may be reused for cross-sectional studies to look at relationships among the study variables, or across days to assess individual differences in behavior.

Specifications table

**Table:** 

Subject	Psychology (General)
Specific subject area	Positive Psychology, Health Psychology
Type of data	Tables
How data were acquired	Self-report through Qualtrics Online Survey Software
Data format	Raw
Parameters for data collection	Criteria for eligibility: at least 18 years old, sober (abstinent) for at least 90 days, and an active member of Alcoholics Anonymous
Description of data collection	In this daily diary study, data were obtained through time-series sampling over 7 days. Participants reported on specific recovery-related behaviors, emotions, and cognitions each evening at 8 pm after receiving a survey link via text message or email.
Data source location	Ann Arbor, Michigan (University of Michigan) United States of America
Data accessibility	Repository name: Mendeley Data Data identification number: 10.17632/4wd9scvdvk. Direct URL to data: https://data.mendeley.com/datasets/4wd9scvdvk/3
Related research article	LaBelle, O. (2020). Daily associations between helping behavior, gratitude, and selfishness in members of Alcoholics Anonymous. *Alcoholism Treatment Quarterly.* https://doi.org/10.1080/07347324.2020.1768992

## Value of the data

• These data provide a novel perspective of the daily behaviors, emotions, and cognitions associated with people in both short-term and long-term recovery from alcohol use disorder.
• Researchers interested in recovery from addiction, recovery processes, personality factors associated with recovery, and who are interested in daily diary and time-series sampling will be interested in this data.
• The data can be used to investigate individual differences in helping behavior, gratitude, hypersensitive narcissism, self-absorption, self-efficacy, social contact, exercise habits, positive/negative affect, and physical activity over a time period of one week. Additionally, measures that assess addiction severity, adult attachment, gratitude, resentment, personality, psychological distress, altruism, social desirability, recovery capital, alcohol-related God locus of control, self-esteem, narcissism, health, spirituality, and religiosity, were collected at baseline.
• The dataset includes open-text answers submitted each night about the high point and low point of the day that could be analyzed qualitatively.

## 1 Data description

The accompanying SPSS Statistics data file contains 904 columns of the self-report responses of 113 participants. Table 1 lists demographic variables collected with their labels, while Table 2 lists recovery-related variables and labels. An overview of the measures from the baseline survey and the daily surveys can be found in Tables 3 and 4, respectively.

**Table 1:** *Main Demographic Variables*.

Column Number	Variable Name	Variable Label	Variable Values
10	D_Age	Age	Open
11	D_Gen	Gender	1 = “Male” 2 = “Female” 3 = “Other (details optional)”
12	D_Race	Race/Ethnicity	1 = “Alaskan Native/Native American” 2 = “Asian/Pac Islander” 3 = “Black/African” 4 = “Hispanic/Latino/a” 5 = “Middle Eastern” 6 = “White/Caucasian” 7 = “Other” 8 = “Bi/Multiracial”
14	D_Ed	What is the highest level of education you have attained?	1 = “Some high school” 2 = “GED/High school diploma” 3 = “Some college” 4 = “College degree” (BA, BS) 5 = “Master's degree (MA, MS, MSW)” 6 = “Professional degree (JD, MBA)” 7 = “Doctorate (MD, PhD)”
15	D_Inc	What is your average annual income?	1 = “0–25,000″ 2 = “25,000 to 50,000″ 3 = “50,000 to 75,000″ 4 = “75,000–100,000″ 5 = “100,00+”
16	D_Rel	What is your relationship status?	1 = “Single” 2 = “Dating” 3 = “In a relationship” 4 = “Married” 5 = “Separated or divorced” 6 = “Widowed”
17	D_Health	In general, how would you rate your health?	1 = “Excellent” 2 = “Very Good” 3 = “Good” 4 = “Fair” 5 = “Poor”
18	D_Emp	Are you employed?	1 = “Yes, full time” 2 = “No” 3 = “Yes, part time”

*Note.* Additional demographic information has been omitted to save space. See full dataset for all available variables*.*

**Table 2:** *Recovery-Related Variables*.

Column Number	Variable Name	Variable Label	Variable Values
26	Q_SobD	What is your sobriety date? (mm/dd/yyyy format)	Open
27	D_Add	What was your main addiction?	1 = “Alcohol” 2 = “Drugs”
30	AA_12S	Are you working the 12-steps with your sponsor?	1 = “Yes” 2 = “No”
31	AA_Mtgs_Est	Number of Weekly Meetings	Open
32	AA_SpOth	Are you sponsoring anyone?	1 = “Yes” 2 = “No”
33	AA_NumSp	Number of Sponsees	Open

**Table 3:** *Overview of baseline measures*.

Column Number	Variable Prefix	Measure	Study Variable	Total Items	Author(s)
34–50	SIP_	Short Inventory of Problems Scale – Revised^b^	Addiction severity	17	*Kiluk* et al.*, 2013*
50–62	ECR_	Experiences in Close Relationships Scale^e^	Adult attachment style (anxious and avoidant)	12	*Brennan* et al.*, 1998*
63–75	NPI_	Narcissistic Personality Inventory^a^	Narcissism	13	*Gentile* et al.*, 2013*
76–95	APS_	Altruistic Personality Scale^c^	Altruism	20	*Rushton* et al.*, 1981*
96–111	GRAT_	Gratitude, Resentment and Appreciation Test^e^	Trait-level gratitude	16	*Watkins* et al.*, 2003*
112–121	TIPI_	Ten-Item Personality Inventory^e^	Big-5 personality traits	10	*Gosling* et al.*, 2003*
122–127	K_6_	Kessler Psychological Distress Scale^c^	Psychological distress	6	*Kessler* et al.*, 2002*
128–137	SDS_	Social Desirability Scale^a^	Social desirability	10	*Strahan & Gerbasi, 1972*
138–149	AGLOC_	Alcohol-God Locus of Control Scale^d^	Locus of control (over alcoholism)	12	*Murray* et al.*, 2006*
150–159	RSE_	Rosenberg Self-Esteem Scale^b^	Self-esteem	10	*Rosenberg, 1965*
160–169	BARC_	Brief Assessment of Recovery Capital^d^	Recovery capital	10	*Vilsaint* et al.*, 2017*

*Notes*.

a Binary scale.

b 4-point Likert-type scale.

c 5-point Likert-type scale.

d 6-point Likert-type scale.

e 7-point Likert-type scale.

**Table 4:** *Overview of the Daily Measures*.

Variable Prefix	Measure	Study Variable	Total Items	Author
SOS_	Service to Others in Sobriety Scale^a^	Helping behavior	12	*Pagano* et al.*, 2010*
GAC_	Gratitude Adjectives Checklist^c^	State-level gratitude	3	*Emmons & McCullough, 2003*
SAS_	Self-Absorption Scale^c^	Private self-absorption Public self-absorption	17	*McKenzie & Hoyle, 2008*
HSNS_	Hypersensitive Narcissism Scale^c^	Covert narcissism	12	*Hendin & Cheek, 1997*
PANAS_	Positive and Negative Affect Schedule^c^	Mood	10	*Watson* et al.*, 1988*
SE_	Self-Efficacy Scale^b^	Self-efficacy	10	*Rosenberg, 1965*

*Notes*.

a Binary scale.

b 4-point Likert-type scale.

c 5-point Likert-type scale.

## 2 Experimental design, materials, and methods

### 2.1 Participants

The sample consisted of 113 members of Alcoholics Anonymous; full demographic information is presented in Table 5, and recovery-related information is presented in Table 6. Across all participants, the length of time in recovery ranged from 5 months to 39 years (M = 7.63 years, SD = 9.17).

**Table 5:** *Sample Demographics*.

Variable	*N*	Valid%
*Age (years)*		
<20	1	0.9
20–29	36	32.1
30–39	42	33.9
40–49	18	16.1
50–59	7	6.3
60–69	7	6.3
70+	1	0.9
*Gender*		
Male	36	31.9
Female	77	68.1
*Race*		
Alaskan Native/Native American	1	0.9
Asian/Pacific Islander	2	1.8
Black/African	1	0.9
Hispanic/Latino/a	4	3.5
White/Caucasian	102	90.3
Bi/Multiracial	3	2.7
*Highest Level of Education*		
Some High School	1	0.9
GED/High School Diploma	2	1.8
Some college	45	39.8
College degree	38	33.6
Master's Degree	20	17.7
Professional Degree	5	4.4
M.D./PhD	2	1.8
*Average Annual Income*		
$0 - $24,999	32	28.3
$25,000 - $49,999	38	33.6
$50,000 – $74,999	27	23.9
$75,000 - $99,999	5	4.4
$100,000+	11	9.7

*Note.* Additional demographic information has been omitted to save space.

See full dataset for all available variables*.*

**Table 6:** *Recovery Related Information*.

Variable	n	Valid%	*M*	*SD*
*Time in Recovery*			7.63	9.17
*Primary Addiction*				
Alcohol	72	63.7		
Drugs	41	36.3		
*Number of Weekly Meetings Attended*			2.99	1.82
0	6	5.4		
1	17	15.1		
2	28	24.8		
3	27	23.9		
4	13	11.5		
5	12	10.6		
6	5	4.4		
7+	5	4.5		
*Working 12-Steps with a Sponsor*				
Yes	101	89.4		
No	11	9.7		
*Sponsoring Others*				
Yes	60	53.1		
No	53	46.9		
*Number of Sponsees*			1.5	1.71

*Note. N* = 113; *M* and *SD* represent mean and standard deviation, respectively.

### 2.2 Materials

The survey was created and administered to participants through the Qualtrics platform. Informed consent was obtained electronically prior to participation. Anonymity was ensured through the assignment of unique identifiers at the time of consent. The participant was required to enter this identification number to complete each daily survey to enable linking daily data to baseline measures while maintaining participant anonymity.

### 2.3 Baseline measures

*Short Inventory of Problems – Revised (SIP-R; Kiluk* et al.*, 2013* [1]*).* A 17-item scale that is designed to assess negative consequences of substance use on a 4-point Likert-type scale.

*Experiences in Close Relationships Scale – Short Form (ECR-S; Wei* et al.*, 2007* [2]*).* A 12-item scale that measures avoidant and anxious subscales of attachment using a 7-point scale.

*Narcissistic Personality Scale - 13 (NPI-13; Gentile* et al.*, 2013* [3]*).* A 13-item scale that uses binary items to measure grandiose narcissism.

*Altruistic Personality Scale (APS; Rushton* et al.*, 1981* [4]). A 20-item scale used to measure baseline trait-level lifetime altruistic behavior using a 5-point scale.

*Gratitude, Resentment and Appreciation Test (GRAT; Watkins* et al.*, 2003* [5]). A 16-item scale used to measure trait gratitude levels on a 9-point scale.

*Ten-Item Personality Inventory (TIPI; Gosling* et al.*, 2003* [6]*).* A 10-item assessment of the big 5 personality traits: was used to assess big 5 personality traits: Openness, Conscientiousness, Extraversion, Agreeableness, and Neuroticism.

*Kessler Non-Specific Psychological Distress Scale (K-6; Kessler* et al.*, 2002* [7]*).* A 6-item measure of distress experienced over a period of 30 days as indicated by responses on a 5-point scale.

*Social Desirability Scale - Short version (SDS; Strahan & Gerbasi,* [8]*).* A scale containing 10 true or false items to measure the participant's tendency to respond in a socially acceptable manner.

*Alcohol-God Locus of Control Scale (AGLOC; Murray* et al.*, 2006* [9]*).* A measure of God/Higher Power control beliefs over alcoholism, calculated using a 6-point Likert response format.

*Rosenberg Self-Esteem Scale (RSE; Rosenberg, 1965* [10]*).* A 10-item, 4-point scale that measures global self-worth by evaluating both positive and negative feelings about the self.

*Brief Assessment of Recovery Capital (BARC-10; Vilsaint* et al.*, 2017* [11]*.* A 10-item measure designed to quantify internal and external resources available to individuals initiating and sustaining recovery that is measured on a 6-point scale.

### 2.4 Daily measures

*Service to Others in Sobriety Scale (SOS; Pagano* et al.*, 2010* [12]*).* A 12-item measure of the frequency of prosocial behavior on that day indicated by yes/no responses. Modified for daily use.

*Gratitude Adjectives Checklist (GAC; Emmons & McCullough, 2003* [13]*). A* 3-item measure commonly used to assess participant's level of daily gratitude as indicated by scores on a 5-point scale.

*The Hypersensitive Narcissism Scale (HSNS; Hendin & Cheek, 1997* [14]*).* A measure of covert narcissism reported on a 5-point Likert scale.

*Self-Absorption Scale (SAS; McKenzie & Hoyle, 2008* [15]*).* A 17-item, 5-point measure of how much time was spent thinking about the self.

*The Positive and Negative Affect Schedule (PANAS; Watson* et al.*, 1988* [16]*).* A 10-item measure of daily mood as indicated by participant response on a 5-point scale.

## 3 Procedure

Recruitment of members of Alcoholics Anonymous was conducted online through advertisements on social media sites (i.e., Twitter, Facebook), the use of recovery-related e-mail listservs, and, subsequently, snowball sampling. Participants who agreed to be contacted regarding future studies related to recovery were also contacted via e-mail (i.e., Life in Recovery Study 1 and 2; LaBelle & Edelstein, 2018 [17]). To be eligible for inclusion, respondents had to be at least 18 years of age, an active member of AA (self-defined) with a sponsor and a minimum of 90 days of sobriety. During recruitment, participants were advised they would be compensated for their time with a single payment of up to $20; The total amount of compensation was calculated based on the receipt of $15 for initial survey completion and an additional bonus payment of $5 for the completion of all seven days of daily reporting. Participants received and responded to the baseline survey via their laptop or desktop computer; daily report surveys could be completed on any device but were optimized for completion on a mobile phone for ease in reporting.

To ensure that the optimal sample size had been achieved, daily reporting commenced five days following the initial survey launch and baseline data collection. Over the following seven days, participants were asked to complete a 10-minute survey each evening, which required them to reflect on prosocial behavior, mood, gratitude, selfishness and basic daily health behaviors. To increase the response rate, email and text message reminders containing a survey link were sent to participants each evening at 8 pm in each participant’s designated time zone. Response rates to the daily survey ranged between 72.6% to 92.9% each night (*M* = 20.1%).

The analyses used in the resultant article was multilevel modeling with daily reports nested within individuals (LaBelle, 2020 [18]). These data may be reused for cross-sectional studies to look at relationships among the study variables, or across days to assess individual differences in behavior.

## CRediT authorship contribution statement

**Onawa LaBelle:** Conceptualization, Methodology, Investigation, Formal analysis, Writing - review & editing, Funding acquisition. **Maurissa Hastings:** Writing - original draft, Visualization, Data curation.

## Declaration of Competing Interest

The authors declare that they have no known competing financial interests or personal relationships which have, or could be perceived to have, influenced the work reported in this article.

## Appendix Supplementary materials

Image, application 1
